# Hürthle cell‐predominant thyroid fine needle aspiration cytology: A four risk‐factor model highly accurate in excluding malignancy and predicting neoplasm

**DOI:** 10.1002/dc.25000

**Published:** 2022-06-08

**Authors:** Lisi Yuan, Christian Nasr, James F. Bena, Tarik M. Elsheikh

**Affiliations:** ^1^ Department of Pathology and Laboratory Medicine Henry Ford Hospital Detroit Michigan USA; ^2^ Department of Endocrinology Diabetes and Metabolism, Cleveland Clinic Cleveland Ohio USA; ^3^ Department of Quantitative Health Sciences Lerner Research Institute, Cleveland Clinic Ohio USA; ^4^ Department of Pathology Pathology and Laboratory Medicine Institute, Cleveland Clinic Cleveland Ohio USA

**Keywords:** ATA, Bethesda system, fine needle aspiration, Hürthle cell, Hürthle cell carcinoma, Hürthle‐cell neoplasm, indeterminate, Oncocytic, thyroid cytology, TI‐RADS, ultrasound

## Abstract

**Background:**

Interpretation of Hürthle cell‐predominant cytologies (HCP) is very challenging as a majority is diagnosed as indeterminate. Prior studies have reported various cytologic features to help distinguish non‐neoplastic (NN) from neoplastic and malignant lesions but had contradicting results. Our aim was to identify risk factors predictive of neoplasm and/or malignancy by correlating cytologic features with clinical and ultrasound findings.

**Methods:**

Sixty‐nine HCP cases with surgical follow‐up were identified, including 35 NN, 20 adenomas, and 14 carcinomas. Ultrasound data were recorded utilizing Thyroid Imaging Reporting and Data System (TI‐RADS) and American Thyroid Association (ATA) scoring systems. Sixteen cytologic criteria were evaluated and semi‐quantitatively scored. Data were assessed by univariable, multivariable and stepwise logistic regression analysis; and statistical significance achieved at *P*‐value <0.05.

**Results:**

On univariable analysis, significant predictors of neoplasm were high cellularity, isolated single cells, absent colloid, non‐uniform HC population (anisonucleosis), larger nodule size, and higher ATA score. Large‐cell dysplasia and transgressing blood vessels were not found to be significant factors. Multivariable analysis identified a combination of four risk factors (high cellularity, anisonucleosis, absent colloid, and size ≥2.9 cm) that was associated with neoplasm in 10/11 patients. None of 15 patients with zero or 1 out of 4 risk factors had malignancy or neoplasm on follow‐up. This model also significantly outperformed ATA and TI‐RADS scoring systems.

**Conclusion:**

In the absence of four or three risk factors, the model excluded malignancy and neoplasm in all patients. The presence of all four factors predicted neoplasm and malignancy in 91% and 46% of cases, respectively.

## 1 INTRODUCTION

Hürthle/oncocytic cells (HCs) are a common finding in thyroid fine needle aspiration cytology (FNA) and can be associated with benign and malignant neoplasms, as well as non‐neoplastic (NN) conditions such as oncocytic metaplasia associated with nodular goiter and lymphocytic thyroiditis. Major differential diagnostic considerations include NN disease, Hürthle cell adenoma (HCA), Hürthle cell carcinoma (HCC), and papillary thyroid carcinoma (PTC) with oncocytic features. FNAs showing admixture of HCs and benign non‐HC components such as abundant colloid, many lymphocytes, and/or thyroid normo‐follicular cells are usually diagnosed as NN, pose no diagnostic challenge, and placed in the “benign” category (B‐2) of the Bethesda system for reporting thyroid cytology (TBSRTC).^1^ However, FNAs consisting exclusively or almost exclusively of HCs, that is, HC‐predominant (HCP), are diagnostically challenging for the pathologist, as it is often difficult to distinguish neoplastic from NN nodules. HCP FNAs often fall into one of two indeterminate TBSRTC categories: atypia/follicular lesion of undetermined significance (AUS/FLUS) and follicular neoplasm/suspicious for follicular neoplasm (FN/SFN).^1^, ^2^ Follow‐up studies, however, have shown that risks of malignancy (ROM) associated with HCP are appreciably lower than those of non‐HCP, which could potentially lead to increased number of unnecessary surgeries.^3^

Molecular studies have been utilized in recent years with intended purpose of increasing the predictive power of indeterminate cytologies,^4^ but HCP indeterminate lesions have not been extensively studied.^4^ Reported negative predictive values (NPV) and positive predictive values (PPV) are 94–96% and 40–46%, respectively.^5^, ^6^, ^7^, ^8^, ^9^ The major strength of molecular testing is identifying nodules that have a high likelihood of being benign, but a major limitation is their low specificity which results in significant false‐positive rates. In addition, reflex molecular testing is not performed at many institutions, and an indeterminate cytologic diagnosis may either lead to a repeat FNA with triage for molecular testing or lobectomy following a repeat indeterminate diagnosis.

Few previous studies have attempted to evaluate cytologic features that can help predict neoplasm or malignancy, but there has been limited agreement regarding the usefulness of specific cytologic criteria.^3^, ^10^ This may have been partly due to the small number of cases included in those studies, limited application of statistical analysis, dilution of study cohorts by easily diagnosed B‐2 category aspirates, and inconsistent to absent incorporation of clinical and imaging features.

The Thyroid Imaging Reporting and Data System (TI‐RADS) and the American Thyroid Association (ATA) ultrasound classification systems were recently introduced and are currently widely utilized in the preoperative evaluation of thyroid nodules.^11^, ^12^, ^13^ Some studies have shown that TI‐RADS and ATA scoring systems can be particularly helpful in the management of thyroid nodules with indeterminate cytology.^14^ However, correlation of cytologic features of HCP aspirates with ATA and TI‐RADS scores and clinical features has not been previously reported.

The aim of the current study was to identify a combination of cytologic, ultrasound, and clinical features that would allow us to construct a statistically significant risk‐factor model that can better predict or exclude the presence of neoplasia and/or malignancy in HCP FNAs that are diagnostically challenging, i.e., aspirates that are diagnosed as “indeterminate” by TBSRTC.

## 2 MATERIAL AND METHODS

Under an institutional review board‐approved protocol, archival cytopathology files of Cleveland Clinic were searched to identify all potential HCP thyroid FNAs diagnosed between 1/1/2010 and 12/31/2014. The distribution of thyroid FNAs over that time was: total cases: 12108; non‐diagnostic: 1333 (11%); benign: 8735 (72%); AUS/FLUS: 874 (7%); FN/SFN: 585 (5%); suspicious for malignancy: 227 (2%); Malignant: 354 (3%). Per TBSRTC guidelines,^1^ Hürthle cell type (HCT) was reported as a subtype of FN/SFN (FN/SFN‐HCT), but not as a subtype of AUS/FLUS. FN/SFN‐HCT comprised 28% of FN/SFN cases (166/585) and 1.4% of all thyroid FNAs. AUS/FLUS was searched for reports that mentioned “Hürthle” or “oncocytic” in the diagnosis or comment lines, and that resulted in 23 cases. Of the total retrieved search of HCP nodules with diagnoses of FN/SFN and AUS/FLUS (189 cases), only cases that had available ultrasound imaging and histopathologic correlation were considered for the study (90 cases); slides were available in 69 of those cases. Therefore, the final study cohort consisted of 69 HCP aspirates from 69 patients (age range 27–86, median 61 years). Distribution of cytologic diagnoses, histologic follow‐up, risk of malignancy (ROM), and risk of neoplasia (RON) are shown in Table 1. For this study, AUS/FLUS with HCP is referred to as AUS/FLUS‐HCT. NN was defined as nodular hyperplasia and/or lymphocytic thyroiditis associated with oncocytic metaplasia. Molecular testing was performed on 4 out of 69 cases (Afirma GEC), and all were resulted as “suspicious.” Follow‐up demonstrated 3 HCA and 1 NN. Age, sex, and nodule size were documented, and ultrasound imaging was interpreted by an endocrinologist with extensive expertise in neck ultrasonography and thyroid neoplasia (CN), blinded to FNA diagnoses and surgical outcomes, and data recorded utilizing ATA and TI‐RADS scoring systems.^12^, ^13^

**TABLE 1:** Cyto‐histologic correlation of Hürthle cell‐predominant cases included in the study

Cytologic diagnosis		Surgical pathology follow‐up					
TBSRTC categories	# Cases(%)	NN	HCA	HCC	PTC, oncocytic	ROM (%)	RON (%)
*AUS/FLUS‐HCT*	7 (10)	5	1	0	1	14	29
*FN/SFN‐HCT*	62 (90)	30	19	9	4	22	52
*Totals*	69	35	20	9	5	20	49

Abbreviations: AUS/FLUS‐HCT, atypia/follicular lesion of undetermined significance‐ Hürthle cell type; FN/SFN‐HCT, follicular neoplasm/suspicious for follicular neoplasm‐ Hürthle cell type; HCA, Hürthle cell adenoma; HCC, Hürthle cell carcinoma; NN, Non‐neoplastic; PTC, Papillary thyroid carcinoma; ROM, risk of malignancy; RON, risk of neoplasia; TBSRTC, the Bethesda System for Reporting Thyroid Cytology.

Sixteen previously reported cytologic criteria^15^, ^16^, ^17^, ^18^, ^19^, ^20^, ^21^, ^22^, ^23^ were evaluated in all cases. The cytologic features, their assessment and definitions are presented in Table 2. All criteria were semi‐quantitatively scored concurrently by 2 Cytopathologists (TME, LY), blinded to final cytologic and histologic diagnoses.

**TABLE 2:** Cytologic features evaluated in 69 Hürthle cell‐predominant nodules^a^

Cytologic feature	Evaluation	Definition
Cellularity	Low or high	*Low*: sparse cellularity
		*High*: moderate to marked cellularity
Percentage of Hürthle cells (≥ 90%)^a^	Lower or higher	
Percentage of admixed normo‐follicular cells	Lower or higher than 50%	
Architecture of Hürthle cells	Predominant flat sheets or three‐dimensional groups	
Microfollicles	< 25% or ≥25%	HCs with repetitive microfollicular pattern
Isolated single cells ≥10%	Absence or presence	Discohesive HCs with intact cytoplasm
Uniformity of Hürthle cell population	Predominant uniform vs. non‐uniform population	*Uniform*: HCs of similar size, without significant variation of nuclear shape or size, and without increased N/C ratios. *Non‐uniform* (*anisonucleosis*): HCs with diffuse significant variation in nuclear size (ranging from less than to greater than twice nuclear size variation) and involving >50% of HCs. In contrast to large‐cell dysplasia (LCD), hyperchromasia and/or nuclear irregularities are not required^20^
Small‐cell dysplasia	Absence or presence	Small cells with high N/C ratio (cytoplasmic diameter less than twice nuclear diameter, with often bland appearance)^19^
LCD	Absence or presence	Large cells with at least two times variability in nuclear size, and typically demonstrating hyperchromasia. Prominent nucleoli and/or irregular nuclear outlines may be present.^19^, ^24^ This feature is usually sporadic in distribution, in contrast to the diffuse nature of anisonucleosis
Colloid	Absence or presence	
	If present, further subcategorized as (a) scant vs. abundant, and (b) predominately thin vs. predominately thick	
Lymphocytes	Absence or presence	
	If present further subcategorized as (a) rare or (b) numerous	
Transgressing blood vessels	Absence or presence	Thin delicate capillaries with distinct capillary nuclei intimately associated with loosely cohesive groups/sheets of HCs.^24^
Intracytoplasmic lumens	Absence or presence	Sharply demarcated intracytoplasmic vacuoles that have a tinctorial quality similar to the slide background
PTC‐like nuclear atypia	Absence or presence	Nuclear enlargement with pale/powdery chromatin, and nuclear irregularities and/or grooves
	If present further subcategorized as (a) focal or (b) diffuse	
Cystic changes	Absence or presence	Many background macrophages

Abbreviations: HC, Hürthle cell; LCD, large cell dysplasia; N/C, Nuclear/cytoplasmic.

a All aspirates had >50% Hürthle cells. Great majority of cases (66/69, 96%) had >90% Hürthle cells.

Unordered categorical factors were summarized using frequencies and percentages and were compared between NN, neoplasm and malignant groups using Pearson chi‐square tests or Fisher exact tests when events were rare. Ordered categorical factors were summarized similarly and compared using Wilcoxon rank sum tests. Continuous measures were summarized using means and standard deviations and compared using two‐sample t‐tests. Receiver operating characteristic (ROC) curve analysis was performed to identify the best size cutoff point for neoplasm and malignancy, and to compare the best fit model against ATA and TI‐RADS scoring rules. In multivariable modeling, multicollinearity was checked using variance inflation factors and condition indices. Normo‐follicular cell percentage, lymphocytes, and small‐cell dysplasia were found to be collinear, likely due to their low frequencies and were removed as candidate risk predictors. Multivariable logistic regression models predicting neoplasm or malignancy were fit. Model selection was performed using penalized regression models with variable selection performed with LASSO methods. Analysis was performed using SAS software (version 9.4; Cary, NC). Statistical significance was evaluated based on P‐value <0.05.

## 3 RESULTS

On univariable analysis, HCP aspirates derived from neoplasms (carcinoma and HCA) were significantly less likely to have a uniform cell population, but more likely to have higher cellularity, isolated single cells, absent colloid, larger nodule size, and higher ATA ultrasound malignancy risk levels (Table 3). Malignant nodules were more likely to be of larger size, have isolated single cells, and higher ATA and TI‐RADS ultrasound malignancy risk scores, when compared to benign lesions (NN and HCA) (Table 4). By ROC analysis, a size cutoff of 2.9 cm or larger provided the best prediction for neoplasm, with a sensitivity of 65% and specificity of 80%; this factor was then utilized in subsequent multivariable modeling. The best size cut‐off point for malignancy was also ≥2.9 cm, with a sensitivity of 71%, and a specificity of 66%.

**TABLE 3:** Univariable statistical analysis of predictors of non‐neoplastic versus neoplasm

		Non‐neoplastic (*N* = 35)		Neoplasm (*N* = 34)		
Factor	Total (*N* = 69)	*N*	Statistics	*N*	Statistics	*p*‐value
Age	59.4 ± 12.9	35	59.7 ± 12.4	34	59.0 ± 13.5	0.82^a1^
Gender		35		34		0.073^b^
Male	25 (36.2)		9 (25.7)		16 (47.1)	
Female	44 (63.8)		26 (74.3)		18 (52.9)	
**High cellularity (moderate‐marked)**	45 (65.2)	35	17 (48.6)	34	28 (82.4)	**0.003** ^c^
**Size (cm)**	2.8 ± 1.6	35	2.2 ± 1.3	34	3.5 ± 1.7	**<0.001** ^a1^
**Size ≥2.9 cm**	29 (42.0)	35	7 (20.0)	34	22 (64.7)	**<0.001** ^c^
Hurthle cells >90%	66 (95.7)	35	33 (94.3)	34	33 (97.1)	0.99^d^
Normo‐follicular Cells ≥50%	2 (2.9)	35	2 (5.7)	34	0 (0.00)	0.49^d^
Flat HC sheets	15 (21.7)	35	5 (14.3)	34	10 (29.4)	0.13^c^
**Isolated single cells**	30 (43.5)	35	10 (28.6)	34	20 (58.8)	**0.011** ^c^
**Uniform HC population**	27 (39.1)	35	18 (51.4)	34	9 (26.5)	**0.034** ^c^
Small‐cell dysplasia	3 (4.3)	35	0 (0.00)	34	3 (8.8)	0.11^d^
LCD	20 (29.0)	35	8 (22.9)	34	12 (35.3)	0.25^c^
**Colloid**		35		34		0.003^b^
Absent	47 (68.1)		18 (51.4)		29 (85.3)	
Scant	13 (18.8)		9 (25.7)		4 (11.8)	
Abundant	9 (13.0)		8 (22.9)		1 (2.9)	
Thick	18 (81.8)	17	13 (76.5)	5	5 (100.0)	0.54^d^
**Colloid present (scant/abundant)**	22 (31.9)	35	17 (48.6)	34	5 (14.7)	***0.003*** ^c^
Lymphocytes		35		34		0.99^b^
Absent	61 (88.4)		31 (88.6)		30 (88.2)	
Rare	7 (10.1)		3 (8.6)		4 (11.8)	
Numerous	1 (1.4)		1 (2.9)		0 (0.00)	
Transgressing blood vessels	29 (42.0)	35	14 (40.0)	34	15 (44.1)	0.73^c^
Intracytoplasmic lumina	12 (17.4)	35	4 (11.4)	34	8 (23.5)	0.18^c^
Nuclear atypia: PTC‐like features		35		34		0.65^b^
Absent	58 (84.1)		30 (85.7)		28 (82.4)	
Focal	9 (13.0)		5 (14.3)		4 (11.8)	
Diffuse	2 (2.9)		0 (0.00)		2 (5.9)	
Cystic changes	10 (14.5)	35	6 (17.1)	34	4 (11.8)	0.73^d^
**Malignancy risk by ATA**		35		34		0.006^b^
Very low suspicion risk	21 (30.4)		14 (40.0)		7 (20.6)	
Low suspicion risk	22 (31.9)		14 (40.0)		8 (23.5)	
Intermediate suspicion risk	17 (24.6)		5 (14.3)		12 (35.3)	
High suspicion risk	9 (13.0)		2 (5.7)		7 (20.6)	
TI‐RADS		35		34		0.18^b^
TR2 not suspicious	5 (7.2)		2 (5.7)		3 (8.8)	
TR3 Mildly suspicious	19 (27.5)		12 (34.3)		7 (20.6)	
TR4 Moderately suspicious	29 (42.0)		16 (45.7)		13 (38.2)	
TR5 Highly suspicious	16 (23.2)		5 (14.3)		11 (32.4)	

*Note*: Statistics presented as Mean ± SD, N (column %). Bold italic denotes statistically significant values.

Abbreviation: ATA, American Thyroid Association Imaging scoring system; HC, Hürthle cell; TI‐RADS, Thyroid Imaging Reporting and Data System.

*p*‐values (statistically significant values are in bold).

^a1^ *t*‐test.

^b^ Wilcoxon Rank Sum test.

^c^ Pearson's chi‐square test.

^d^ Fisher's Exact test.

**TABLE 4:** Univariable statistical analysis of predictors of benign versus malignancy

		Benign (*N* = 55)		Malignant (*N* = 14)		
Factor	Total (*N* = 69)	*N*	Statistics	*N*	Statistics	*p*‐value
Age	59.4 ± 12.9	55	58.4 ± 13.1	14	63.1 ± 11.8	0.23^a1^
Gender		55		14		0.57^b^
Male	25 (36.2)		19 (34.5)		6 (42.9)	
Female	44 (63.8)		36 (65.5)		8 (57.1)	
High cellularity	45 (65.2)	55	34 (61.8)	14	11 (78.6)	0.35^d^
**Size (cm)**	2.8 ± 1.6	55	2.5 ± 1.3	14	4.1 ± 2.1	***0.012*** ^a2^
**Size ≥2.9 cm**	29 (42.0)	55	19 (34.5)	14	10 (71.4)	***0.013*** ^c^
Hurthle cells ≥90%	66 (95.7)	55	53 (96.4)	14	13 (92.9)	0.50^d^
Normo‐follicular Cells ≥50%	2 (2.9)	55	2 (3.6)	14	0 (0.00)	0.99^d^
Flat HC sheets	15 (21.7)	55	10 (18.2)	14	5 (35.7)	0.17^d^
**Isolated single cells**	30 (43.5)	55	20 (36.4)	14	10 (71.4)	***0.018*** ^c^
Uniform HC population	27 (39.1)	55	24 (43.6)	14	3 (21.4)	0.13^c^
Small‐cell dysplasia	3 (4.3)	55	3 (5.5)	14	0 (0.00)	0.99^d^
LCD	20 (29.0)	55	16 (29.1)	14	4 (28.6)	0.99^d^
Colloid		55		14		0.094^b^
Absent	47 (68.1)		35 (63.6)		12 (85.7)	
Scant	13 (18.8)		11 (20.0)		2 (14.3)	
Abundant	9 (13.0)		9 (16.4)		0 (0.00)	
Thick	18 (81.8)	20	16 (80.0)	2	2 (100.0)	0.99^d^
Colloid present (scant/abundant)	22 (31.9)	55	20 (36.4)	14	2 (14.3)	0.20^d^
Lymphocytes		55		14		0.23^b^
Absent	61 (88.4)		50 (90.9)		11 (78.6)	
Rare	7 (10.1)		4 (7.3)		3 (21.4)	
Numerous	1 (1.4)		1 (1.8)		0 (0.00)	
Transgressing blood vessels	29 (42.0)	55	23 (41.8)	14	6 (42.9)	0.94^c^
Intracytoplasmic lumina	12 (17.4)	55	9 (16.4)	14	3 (21.4)	0.70^d^
Nuclear atypia: PTC‐like features		55		14		0.51^b^
Absent	58 (84.1)		47 (85.5)		11 (78.6)	
Focal	9 (13.0)		7 (12.7)		2 (14.3)	
Diffuse	2 (2.9)		1 (1.8)		1 (7.1)	
Cystic changes	10 (14.5)	55	8 (14.5)	14	2 (14.3)	0.99^d^
**Malignancy risk by ATA**		55		14		***<0.001*** ^b^
Very low suspicion risk	21 (30.4)		21 (38.2)		0 (0.00)	
Low suspicion risk	22 (31.9)		19 (34.5)		3 (21.4)	
Intermediate suspicion risk	17 (24.6)		13 (23.6)		4 (28.6)	
High suspicion risk	9 (13.0)		2 (3.6)		7 (50.0)	
**TI‐RADS**		55		14		***0.004*** ^b^
TR2 Not Suspicious	5 (7.2)		5 (9.1)		0 (0.00)	
TR3 Mildly suspicious	19 (27.5)		17 (30.9)		2 (14.3)	
TR4 Moderately suspicious	29 (42.0)		25 (45.5)		4 (28.6)	
TR5 Highly suspicious	16 (23.2)		8 (14.5)		8 (57.1)	

*Note*: Statistics presented as Mean ± SD, N (column %).

Abbreviations: ATA, American Thyroid Association Imaging scoring system; HC, Hürthle cell; TI‐RADS, Thyroid Imaging Reporting and Data System.

^a1^ t‐test.

^a2^ Satterthwaite *t*‐test.

^b^ Wilcoxon rank sum test.

^c^ Pearson's chi‐square test.

^d^ Fisher's exact test.

**FIGURE 1:**
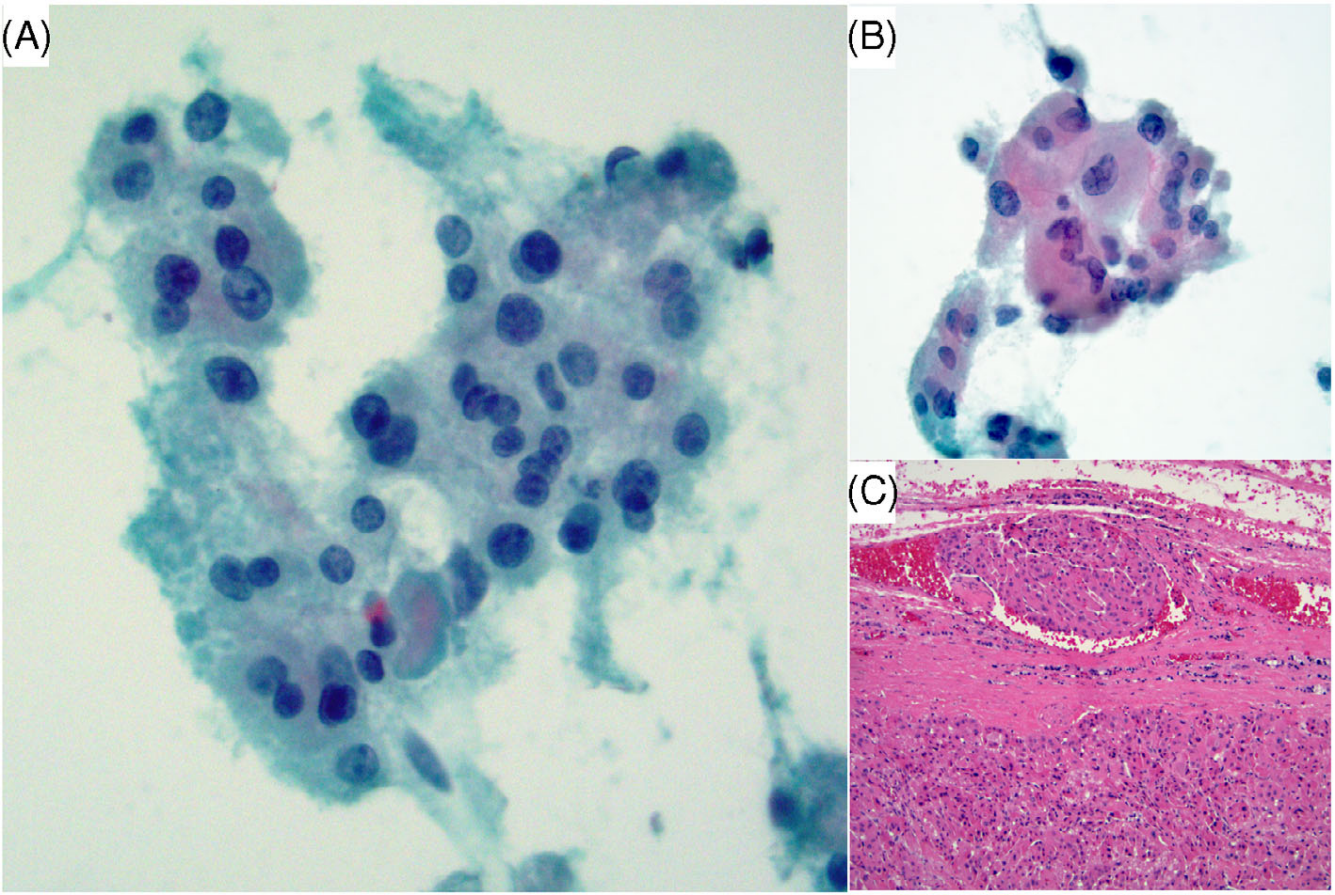
This case had 4 risk factors. (A) FNA of a 3.5 cm nodule with hypercellularity, non‐uniform Hürthle cell population (anisonucleosis), and absent colloid (Papanicolaou stain x 400). (B) There was focal large cell dysplasia (Papanicolaou stain x 400). C) Follow‐up thyroidectomy revealed an angioinvasive Hürthle cell carcinoma (H&E stain x 200) [Colour figure can be viewed at wileyonlinelibrary.com]

**FIGURE 2:**
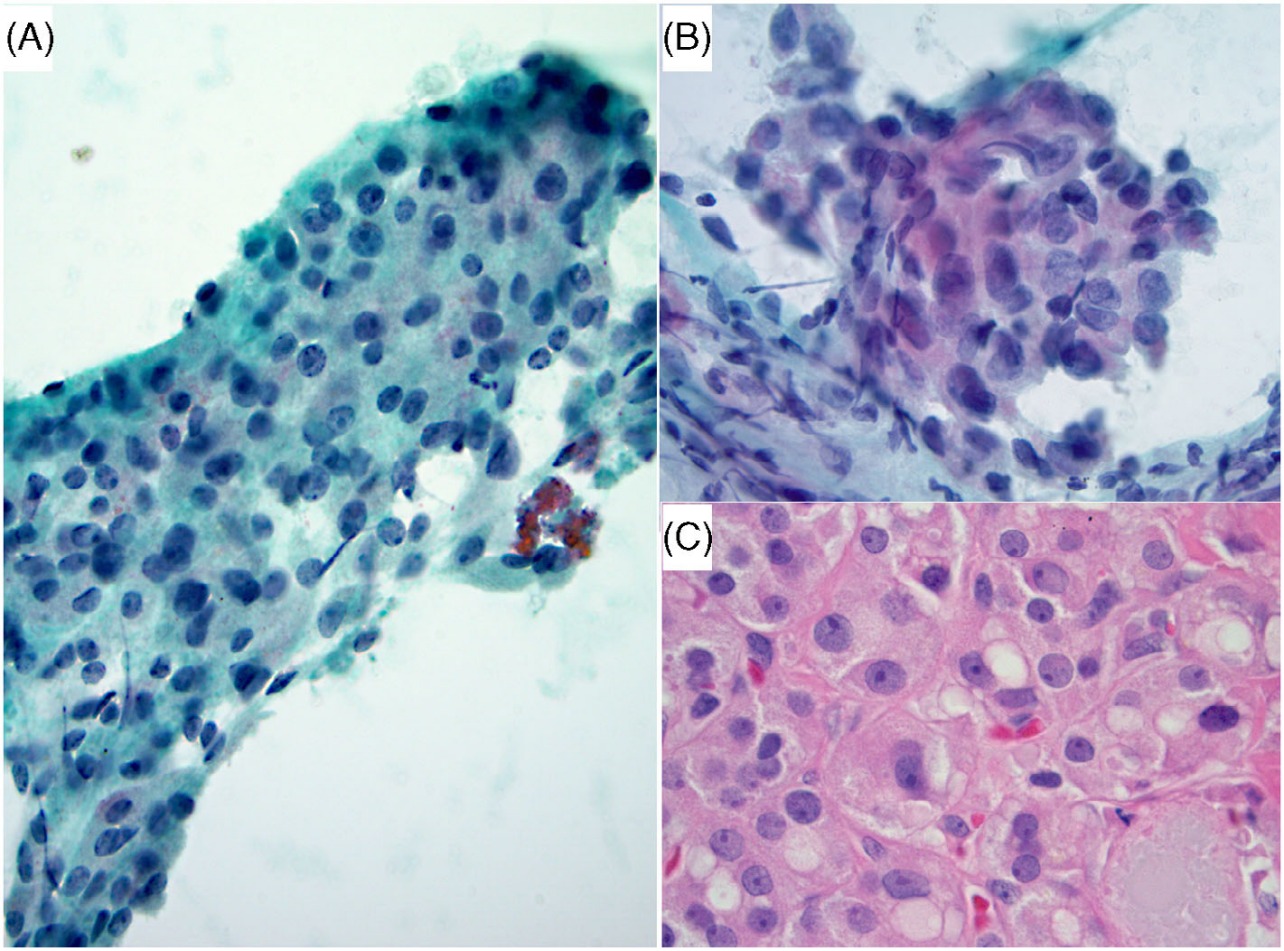
A) This 4.3 cm nodule showed hypercellularity, non‐uniform population, and absent colloid (four risk factors) (Papanicolaou stain x 400). Follow‐up demonstrated Hürthle cell adenoma (HCA) (not shown). (B and C) This nodule was of low cellularity and had 3 risk factors: size of 3.8 cm nodule, non‐uniform population, and absent colloid (not shown). (B) There was focal nuclear irregularity and atypia raising the possibility of papillary thyroid carcinoma (PTC)‐like changes (ThinPrep, Papanicolaou stain x 600). (C) Follow‐up histology revealed a HCA with slight nuclear irregularities, but no evidence of PTC (H&E stain x 600) [Colour figure can be viewed at wileyonlinelibrary.com]

**FIGURE 3:**
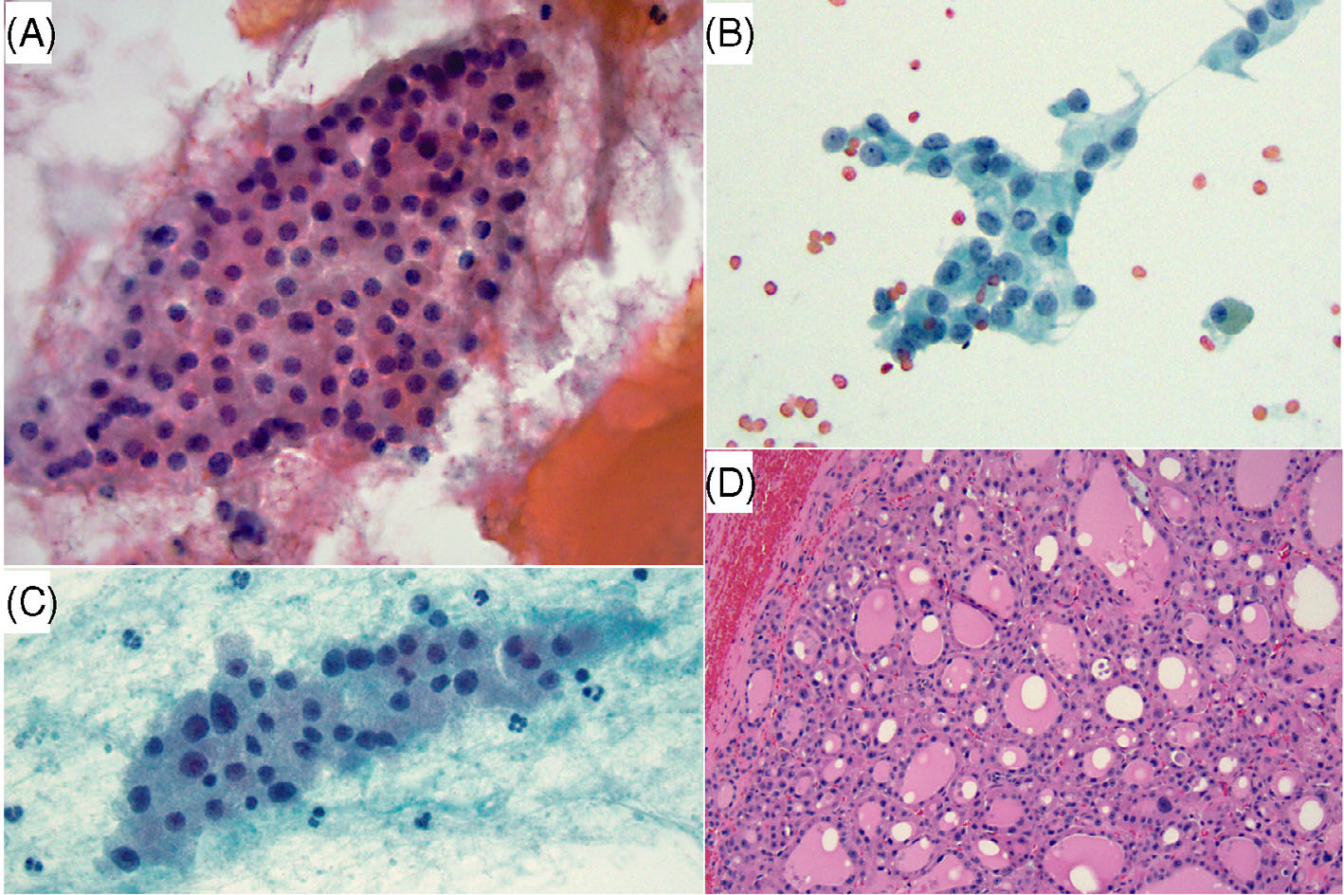
(A) This nodule had zero out of 4 risk factors, including uniform cell population and scant colloid. Cytology was signed out as SFN‐HCT, but histologic follow‐up demonstrated nodular hyperplasia (NH) (not shown). (Papanicolaou stain x 400). (B)–(D): This is another case that had 0/4 risk factors including a predominately uniform Hurthle cell population (B) with only focal/scattered anisonucleosis (C) (Papanicolaou stain x 400). The FNA was signed out as SFN‐HCT. Histologic follow‐up (D) showed NH with random nuclear/endocrine atypia (H&E stain x200) [Colour figure can be viewed at wileyonlinelibrary.com]

**FIGURE 4:**
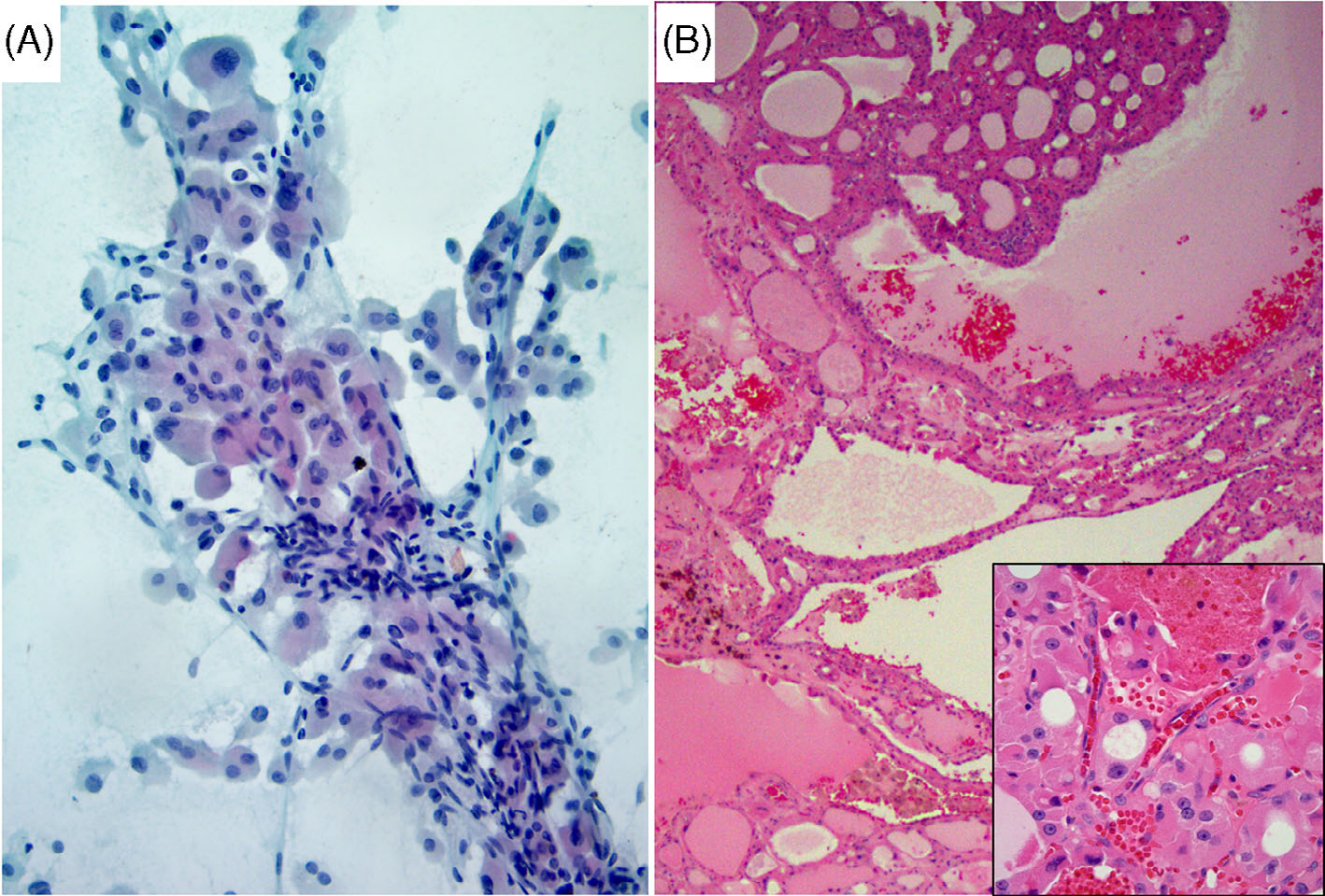
(A) This aspirate had prominent transgressing blood vessels (TBV), large‐cell dysplasia (LCD), and isolated single cells in the background (Papanicolaou stain x 400). The FNA was signed out as SFN‐HCT. (B) Follow‐up histology revealed nodular hyperplasia with oncocytic metaplasia (H&E stain x100). Prominent vascularity within the hyperplastic nodule explains the presence of TBV in the FNA (inset) [Colour figure can be viewed at wileyonlinelibrary.com]

**FIGURE 5:**
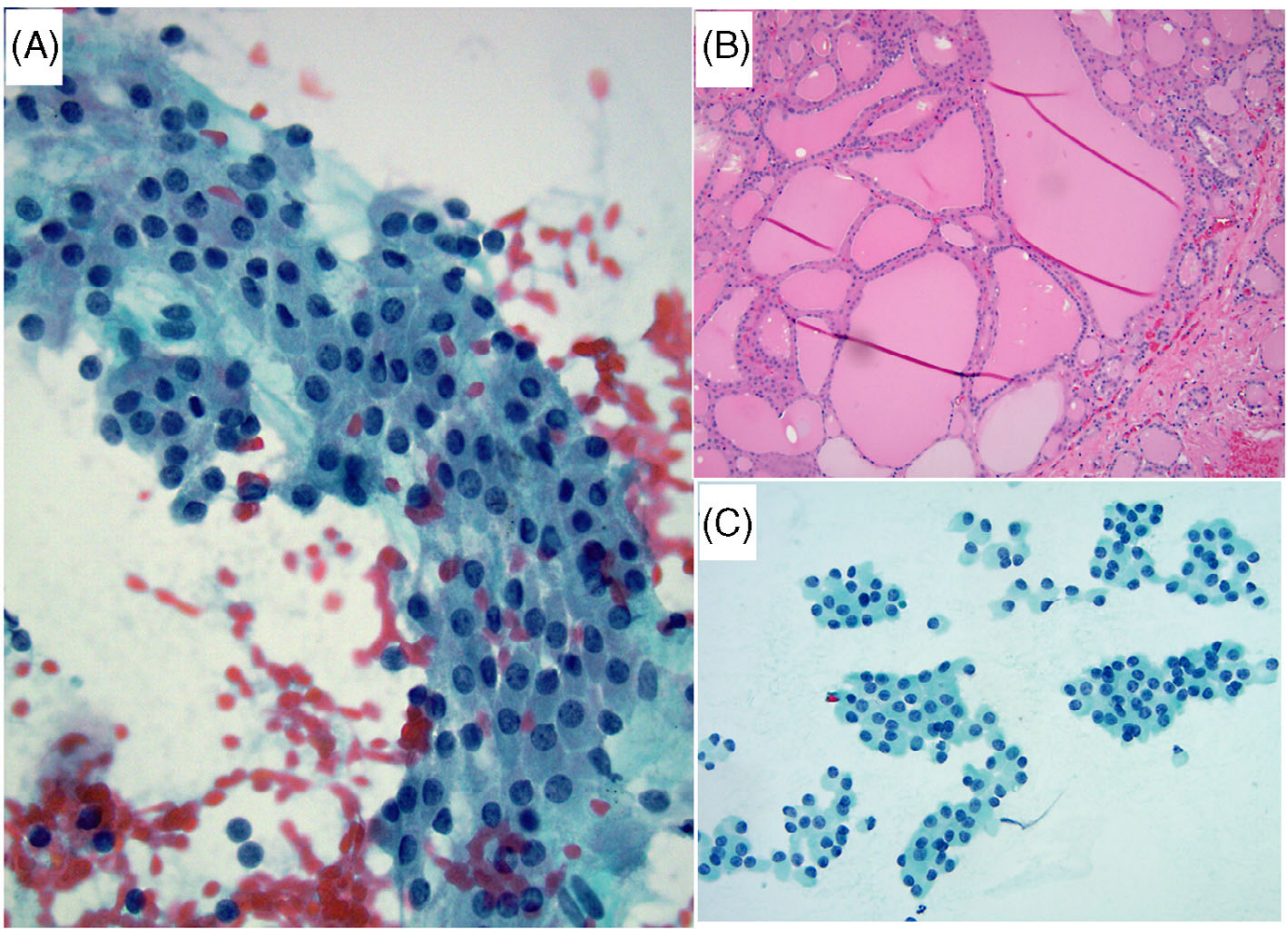
A‐B) This case had one out of 4 risk factors. It's an FNA of a 2.3 cm nodule that was of low (sparse) cellularity and showed uniform Hurthle cell population (A) and absent colloid (Papanicolaou stain x 400). Follow‐up histology revealed a hyperplastic nodule (HN) with oncocytic metaplasia (B). Although this HN had a macrofollicular architecture on histology, there was absent colloid on the corresponding FNA (H&E stain x200). C) This is another FNA where the only risk factor was hypercellularity, as the Hürthle cells had uniform appearance and there was scant colloid present elsewhere (Papanicolaou stain x 200). Cytology was signed out as SFN‐HCT, and histologic follow‐up showed nodular hyperplasia (not shown) [Colour figure can be viewed at wileyonlinelibrary.com]

**FIGURE 6:**
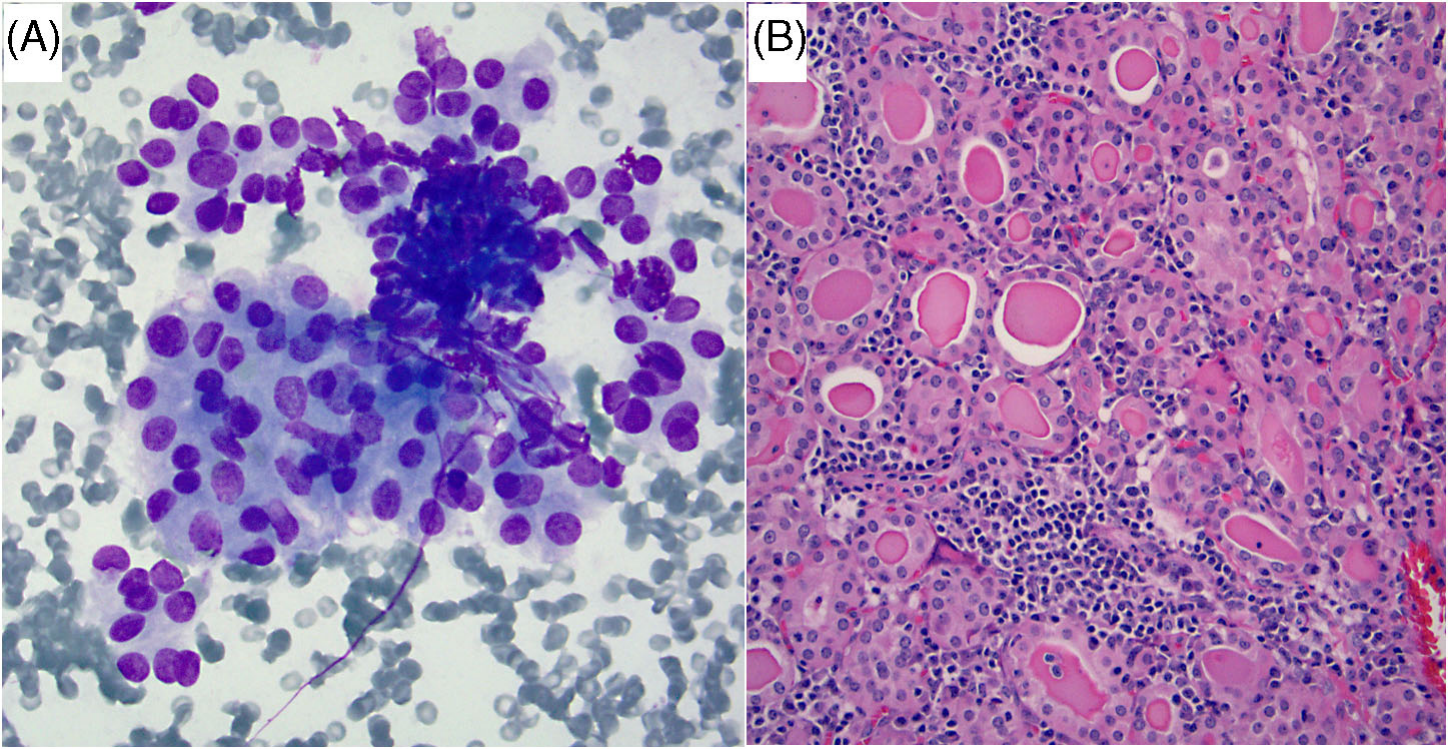
A‐B) This nodule demonstrated only one out of four risk factors: non‐uniform Hurthle cell population, but had low cellularity, scant colloid, and a size of 1.8 cm. (A) There was, however, a prominent microfollicular arrangement and rare background lymphocytes (Diff Quik stain x 400). The cytology was signed out as SFN/FN. (B) Follow‐up lobectomy showed nodular lymphocytic thyroiditis with areas of microfollicular architecture [Colour figure can be viewed at wileyonlinelibrary.com]

Multivariable model selection for neoplasm identified cellularity, nodule size, isolated single cells, uniformity of HC population, and colloid as potential predictors. Given that a predictive model for data this size can only use 3 or 4 effects, further reductions based on level of statistical significance was performed, and a final predictive model based on 4 risk factors was constructed. FNAs with high cellularity, size of ≥2.9 cm, non‐uniform HC population, and absent colloid were at greater risk of being neoplastic (Table 5). This predictive model provided a C‐statistic of 0.88, indicating that the model was successful 88% of the time in assigning a higher risk for neoplasm compared to NN. Ten of 11 patients with all 4 risk factors had neoplasm (RON: 90.9%) (Table 6), and 5 of those had HCC (ROM: 45.5%) (Figures 1 and 2). For malignancy, sensitivity, specificity, PPV, and NPV were 35.7%, 89.1%, 45.5%, and 84.5%, respectively. None of the 3 patients with zero risk factors had neoplasm or malignancy, and none of 12 patients with one risk factor had neoplasm or malignancy (Table 6) (Figures 3, 4, 5, 6). Therefore, in utilizing this model, the absence of 3 or 4 risk factors was associated with sensitivity, specificity, PPV, and NPV of 100%, 27.3%, 25.9%, and 100%, respectively. Compared against ATA and TI‐RADS scoring systems, this four‐risk factor model performed significantly better at predicting neoplasm (Table 7).

**TABLE 5:** Multivariable four‐risk factor model for neoplasm

Risk factor	OR (95% CI)	*p*‐value
Colloid absent	13.38 (2.60, 68.71)	0.002
Size ≥2.9 cm	8.55 (2.14, 34.22)	0.002
Non‐uniform Hurthle cell population	4.01 (1.04, 15.52)	0.044
Cellularity high	6.65 (1.54, 28.67)	0.011

Abbreviations: OR, odds ratio; CI, confidence interval; and *C*‐statistic = 0.879.

**TABLE 6:** Number of risk factors and follow‐up histologic diagnoses

Number of risk factors	Histologic follow‐up				ROM (%)	RON (%)
	Total	Non‐neoplastic	HCA	Malignant		
4/4 risk factors	11	1	5	5	46	91
0/4 risk factors	3	3	0	0	0	0
1/4 risk factors	12	12		0	0	0
0 and 1 risk factor^a^	15	15	0	0	0	0

Abbreviations: HCA, Hürthle cell adenoma; ROM, risk of malignancy; and RON, risk of neoplasia.

^a^ Sum of data for zero and one risk factor (four‐ and three‐risk factors absent).

## 4 DISCUSSION

The cytologic evaluation of HCP FNAs can be quite challenging, as it's often difficult to distinguish NN from HCA or carcinoma. Furthermore, follow‐up studies have shown that ROMs associated with HCP FNAs are appreciably lower than those of non‐HCP (0–30% for AUS‐HCT [median 15%], and 14–45% for FN/SFN‐HCT [median 23%], compared to 10–30% for AUS/FLUS, and 25–40% for FN/SFN.^3^ These lower ROM rates are very similar to those calculated in our cohort study: 14% and 21% for AUS/FLUS‐HCT and FN/SFN‐HCT, respectively (Table 1). Many cytologic features have been previously suggested to distinguish NN from neoplastic and malignant HCP lesions, but no set of criteria has been widely accepted.^21^ Many of those studies evaluated HC‐rich cytologies that included diagnostically non‐challenging B‐2 cases. However, the goal of our study was to identify a more specific combination of cytologic, ultrasound, and clinical features that would help us better predict or exclude the presence of neoplasia and/or malignancy in diagnostically challenging cases that were diagnosed as “indeterminate” by TBSRTC.

We correlated 16 previously reported cytomorphologic features with clinical parameters and ATA and TI‐RADS ultrasound scoring systems, individually and combined, and with surgical outcome. We then constructed predictive models for neoplasia and malignancy based on univariable and multivariable stepwise logistic regression analysis of statistically significant cytologic, clinical and ultrasound risk factors. The combination of 4 features: high cellularity (moderate to marked), non‐uniform HC population (anisonucleosis), absence of colloid, and nodule size of ≥2.9 cm was found to be a much better predictor of neoplasm and malignancy than has been previously reported.^3^ Utilizing this four‐risk factor model, ROM and RON for indeterminate diagnoses (combined AUS/FLUS‐HCT and FN/SFN‐HCT) improved from 20% to 46% and 49% to 91%, respectively (Tables 1, 6). Even more impressive, was the model's ability to exclude malignancy and neoplasm in the absence of those factors. None of 15 patients with zero or one out of four risk factors, that is, the absence of four or three risk factors, had malignancy or neoplasm on surgical follow‐up (Table 6) (Figures 3, 5, 6), resulting in 0% ROM (0/15) and 0% RON (0/15).

**TABLE 7:** Comparison of four‐risk factor model to ultrasound imaging scoring systems in predicting neoplasm

	AUC (95% CI)	*P*‐value (vs. four‐risk factor model)
Four‐risk factor model	0.879 (0.799, 0.960)	N/A
ATA	0.690 (0.568, 0.812)	0.016
TI‐RADS	0.590 (0.461, 0.720)	<0.001

Abbreviations: ATA, American Thyroid Association ultrasound classification system; AUC, area under curve; CI, confidence interval; N/A, Not applicable; TI‐RADS, thyroid imaging reporting and data systems.

According to the thyroid Bethesda book, HCP FNA is diagnosed as FN/SFN‐HCT if it's highly cellular with additional supporting features such as little or no colloid, rare or absent lymphocytes, isolated single cells or three‐dimensional (3‐D) groups, dysplasia, and transgressing blood vessels (TBV).^24^ HCP aspirates of low cellularity and minimal colloid, on the other hand, were recommended to be diagnosed as AUS/FLUS.^25^ In an elegant study, constructed in a similar fashion to ours but only evaluated cytologic features, Elliott et al. reported that the combination of absent colloid, absent chronic inflammation, non‐macrofollicular architecture (single isolated cells, microfollicles, or 3‐D groups), and TBV correctly identified HC neoplasms in 86% of their cases.^16^ Some authors reported that anisonucleosis, hypercellularity, and absent colloid, among other features, were associated with neoplasia and malignancy.^15^, ^16^, ^17^, ^18^, ^19^, ^20^, ^21^, ^23^, ^26^ Our data corroborated some of the above‐listed criteria, but did not support other criteria previously cited to be diagnostic of neoplasia or malignancy. Although isolated single cells were found to be associated with neoplasia in some studies,^16^, ^18^, ^22^, ^23^, ^27^ we demonstrated this feature to only have significant association with neoplasia and malignancy in univariable analyses and not on multivariable analyses, similar to Elliott et al findings.^16^ The presence of abundant colloid and many lymphocytes in HCP aspirates have been previously shown to favor NN, including nodular goiter and lymphocytic thyroiditis.^16^, ^22^ Our study found the presence of abundant colloid to be associated with NN nodules and benign neoplasm, but no statistical significance was achieved due to small number of cases with abundant colloid (9/69). This is explained by the fact that our study only evaluated indeterminate cytologies, and cases containing abundant colloid most likely were signed out as benign (B‐2). However, the absence of colloid was significantly associated with neoplasm (Table 3). There was no significant association between the presence of lymphocytes and the neoplastic or NN nature of the aspirated nodules. Most of our study cohort (61/69 cases), however, had rare or absent lymphocytes in the background, and only one case had numerous lymphocytes (Table 3), limiting our ability to fully evaluate this criterion. However, this is also explained by our study focusing on indeterminate categories B‐3 and B‐4, suggesting that most HCP FNAs with numerous lymphocytes were signed out as “B‐2” at our institution, and thus were not included in the study population.

Several studies described that HCP aspirates lacking both small‐cell dysplasia and large‐cell dysplasia (LCD) are almost never malignant,^19^, ^20^, ^22^ and those with either small‐cell dysplasia or LCD or isolated single cells are associated with HCC.^19^, ^22^ The Bethesda book listed small‐cell dysplasia and LCD as important criteria for FN/SFN‐ HCT, although mentioned that dysplasia (particularly LCD), by itself, is an unreliable feature.^24^ Renshaw et al., on the other hand, stated that utilizing anisonucleosis as a criterion, rather than LCD, increased FNA sensitivity for HCC.^20^ We agree with the latter statement that anisonucleosis (non‐uniform cell population) has more significance than LCD. Our data showed that LCD was not a significant factor (Figures 1 and 4), as it was expressed at similar rates in malignant (29%) and benign nodules (29%) (Table 4); and at slightly lower but not statistically significantly different rates in NN (24%) compared to neoplasm (34%) (Table 3). Due to the low frequency of small‐cell dysplasia in our series (3/69 cases), its significance could not be fully evaluated. Intracytoplasmic lumens (ICL) and TBV have been reported to be associated with neoplasms, and particularly the presence of TBV to be strongly supportive of neoplasm over NN.^24^ Yang et al., reported that TBV was only found in HC neoplasms, and that ICL was detected in 70% of neoplasms.^28^ But others considered TBV and ICL, in addition to microfollicular arrangement, isolated single cells, small‐cell dysplasia and LCD to be non‐specific.^23^ Our data demonstrated TBV to be a nonspecific criterion, as it was present in similar proportions in NN vs. neoplastic lesions (41% vs. 43%), and in benign vs. malignant lesions (42% vs. 43%) (Tables 3, 4). Many of the NN cases on histologic resection showed areas of hypervascularity within the oncocytic hyperplastic nodules, explaining the presence of TBV on corresponding FNAs (Figure 4). ICL were seen in 12% and 23% of NN and neoplastic nodules, respectively; and in 16% and 21% of benign and malignant lesions, respectively, also establishing it as a nonspecific feature (Tables 3, 4).

Increased amount of eosinophilic cytoplasm is often encountered in PTC, which may mimic HC lesions in FNAs. PTC can be readily recognized if PTC nuclear atypia is overt and well‐developed, but if it's subtle it may be underdiagnosed as AUS/FLUS‐HCT or FN/SFN‐HCT. This may explain why PTCs have been reported in 25–44% of histologically resected malignant HCP lesions.^29^, ^30^, ^31^ In our study, oncocytic variant of PTC accounted for 36% (5/14) of histologically confirmed malignant nodules (Table 1). Despite that, we found PTC‐like nuclear atypia, including powdery chromatin, nuclear enlargement and irregularity, and grooves to be of little significance in distinguishing NN from neoplastic, benign from malignant, and PTC from HCC (Figure 2 B‐C), especially when the atypia is focally present (Tables 3, 4). This is probably explained by the fact that HCs in benign conditions can demonstrate various degrees of nuclear atypia and irregularities mimicking PTC.^24^

Nodule size appeared to have a significant association with neoplasia and malignancy in several reports.^22^, ^29^, ^32^, ^33^, ^34^, ^35^, ^36^ In our study, the best size cutoff points for neoplasm and malignancy was ≥2.9 cm, similar to Elliott et al. observations of 2.9 cm as the mean size of HC neoplasms.^16^ Guerrero et al found a nodule size of ≥4 cm to have a 55% association with malignancy,^37^ while Lee et al suggested tumor size of ≥2.5 cm, hypoechoic nodule and malignant ultrasound features to be predictive factors of malignancy in FN/SFN‐HCT.^34^ TI‐RADS and ATA thyroid ultrasound scoring systems were recently introduced and are commonly used classifications.^14^ In a recent study of 323 nodules, ATA and TI‐RADS provided similar diagnostic performances in predicting cancer, including a sensitivity of 77–78%, specificity of 73–76%, PPV of 52–55%, and NPV of 90%.^38^, ^39^ However, A recent meta‐analysis comparing various thyroid ultrasound imaging classifications found a higher performance of TI‐RADS in selecting thyroid nodules for FNA.^40^ Unfortunately, we could not find significant literature addressing ATA and TIRADS ultrasound findings in HCP indeterminate lesions. In our study, ATA risk levels and TI‐RADS scores achieved significant associations with neoplasm and malignancy only on univariable analyses. Comparison of the two ultrasound scoring systems demonstrated ATA to be more useful in predicting neoplasia and malignancy, while TI‐RADS provided acceptable performance in identifying malignancy. However, our four‐risk factor model significantly outperformed both ultrasound scoring systems in predicting neoplasia (Table 7). Some authors reported that patients with carcinoma were typically older, and/or that males carried a higher risk of malignancy,^22^, ^29^, ^30^, ^41^, ^42^ while others reported no association between age^32^, ^43^ or sex^29^, ^32^, ^44^ with RON or ROM. Our data found no statistically significant relationship between age and sex with neoplasia or malignancy.

Finally, there are several potential limitations to our study. First, our analyses were based on a single cohort from a single institution, therefore introducing potential institutional referral and assessment biases. Second, we only evaluated cases that had surgical follow‐up, and there may have been additional clinical features that warranted the resection of these nodules, and potentially influencing RON and ROM. Third, cytologic criteria such as small‐cell dysplasia was removed from our analyses as candidate risk predictors due to its low frequency; therefore, its significance as a criterion could not be fully evaluated. Fourth, at our institution, per TBSRTC guidelines, B‐3 diagnoses were not subclassified, so we may have not captured all AUS/FLUS‐HCT in our cohort since we only retrieved cases that listed “HC” component in the comment or diagnosis lines.

In summary, we identified a combination of 4 features to be strong excluders or predictors of neoplasm and malignancy: nodule size ≥2.9 cm, hypercellularity, non‐uniform HC population (anisonucleosis), and absence of colloid. This model was powerful in excluding neoplasia and malignancy, as the absence of 3 or 4 risk factors established an NPV of 100%. The presence of all 4 factors was associated with a ROM of 46% and RON of 91%, which is a substantial improvement over ROM and RON associated with cytologic diagnoses alone. We also found LCD and TGBV to be non‐significant factors in discriminating NN from neoplasm and malignancy. Furthermore, this four‐risk factor model significantly outperformed other individual or combined clinical and cytologic features and ATA and TI‐RADS ultrasound scoring systems. Additional studies are recommended to further validate these findings.

## AUTHOR CONTRIBUTIONS

Lisi Yuan contributed to data acquisition, data analysis and interpretation, and manuscript writing and editing. Christian Nasr contributed to data acquisition and manuscript editing. James F. Bena performed statistical analysis. Tarik M. Elsheikh contributed to study conception, data acquisition, data interpretation, and manuscript writing and editing.

## Data Availability

The data that support the findings of this study are available on request from the corresponding author. The data are not publicly available due to privacy or ethical restrictions.
